# Zwitterionic (*E*)-1-[(4-nitro­phen­yl)iminio­meth­yl]naphthalen-2-olate

**DOI:** 10.1107/S1600536811012359

**Published:** 2011-04-13

**Authors:** Maamar Damous, Meriem Hamlaoui, Sofiane Bouacida, Hocine Merazig, Jean-Claude Daran

**Affiliations:** a Unité de Recherche de Chimie de l’Environnement et Moléculaire Structurale, CHEMS, Université Mentouri-Constantine, 25000 Algeria; b Département Sciences de la Matière, Faculté des Sciences Exactes et Sciences de la Nature et de la Vie, Université Larbi Ben M’hidi, Oum El Bouaghi 04000, Algeria; c Laboratoire de Chimie de Coordination, UPR CNRS 8241, 205 route de Narbonne, 31077 Toulouse Cedex, France

## Abstract

The title compound, C_17_H_12_N_2_O_3_, was synthesized by the reaction of 2-hy­droxy-1-naphthaldehyde with 4-nitro­benzenamine. These condense to form the Schiff base, which crystallizes in the zwitterionic form. In the structure, the keto–amino tautomer has a fairly short intra­molecular N—H⋯O hydrogen bond between the 2-naphthalenone and amino groups, with electron delocalization. The mol­ecule is essentially planar, with a dihedral angle of 1.96 (3)° between the ring systems. In the crystal, the mol­ecules are linked *via* inter­molecular C—H⋯O hydrogen bonds, forming a layer parallel to (101).

## Related literature

For background to Schiff base compounds, see: Fan *et al.* (2007 ▶); Kim *et al.* (2005 ▶); Nimitsiriwat *et al.* (2004 ▶). For the pharmaceutical and medicinal activity of Schiff bases, see: Chen *et al.* (1997 ▶); Dao *et al.* (2000 ▶); Ren *et al.* (2002 ▶); Sriram *et al.* (2006 ▶); Karthikeyan *et al.* (2006 ▶). For Schiff bases in coordination chemistry, see: Ali *et al.* (2008 ▶); Kargar *et al.* (2009 ▶); Yeap *et al.* (2009 ▶). For related structures, see: Fun *et al.* (2009 ▶); Nadeem *et al.* (2009 ▶); Eltayeb *et al.* (2008 ▶). For standard bond lengths see: Allen, (2002 ▶). 
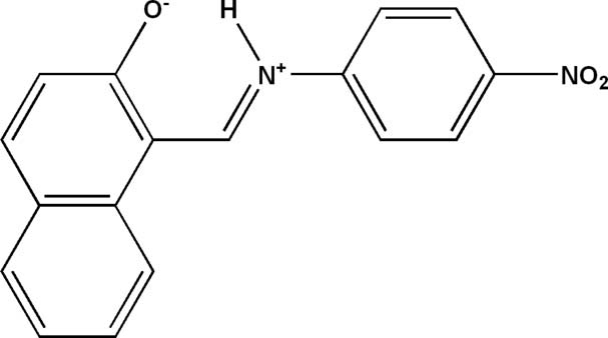


## Experimental

### 

#### Crystal data

C_17_H_12_N_2_O_3_
*M* *_r_* = 292.29
Monoclinic, 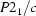

*a* = 8.0503 (6) Å
*b* = 12.8174 (9) Å
*c* = 13.1833 (10) Å
β = 97.271 (5)°
*V* = 1349.37 (17) Å^3^
*Z* = 4
Mo *K*α radiation
μ = 0.10 mm^−1^
*T* = 296 K
0.15 × 0.06 × 0.04 mm

#### Data collection

Bruker SMART CCD area-detector diffractometer
44074 measured reflections
7946 independent reflections
3658 reflections with *I* > 2σ(*I*)
*R*_int_ = 0.074

#### Refinement

*R*[*F* ^2^ > 2σ(*F* ^2^)] = 0.059
*wR*(*F* ^2^) = 0.190
*S* = 0.96
7946 reflections
207 parameters
H atoms treated by a mixture of independent and constrained refinement
Δρ_max_ = 0.53 e Å^−3^
Δρ_min_ = −0.26 e Å^−3^

### 

Data collection: *SMART* (Bruker, 2007 ▶); cell refinement: *SAINT* (Bruker, 2007 ▶); data reduction: *SAINT*; program(s) used to solve structure: *SIR2002* (Burla *et al.*, 2003 ▶); program(s) used to refine structure: *SHELXL97* (Sheldrick, 2008 ▶); molecular graphics: *ORTEP-3 for Windows* (Farrugia, 1997 ▶) and *DIAMOND* (Brandenburg & Berndt, 2001 ▶); software used to prepare material for publication: *WinGX* (Farrugia, 1999 ▶).

## Supplementary Material

Crystal structure: contains datablocks global, I. DOI: 10.1107/S1600536811012359/bq2290sup1.cif

Structure factors: contains datablocks I. DOI: 10.1107/S1600536811012359/bq2290Isup2.hkl

Additional supplementary materials:  crystallographic information; 3D view; checkCIF report

## Figures and Tables

**Table 1:** Hydrogen-bond geometry (Å, °)

*D*—H⋯*A*	*D*—H	H⋯*A*	*D*⋯*A*	*D*—H⋯*A*
N2—H2*N*⋯O3	1.09 (2)	1.57 (2)	2.5287 (15)	143 (2)
C5—H5⋯O2^i^	0.93	2.59	3.5136 (16)	173
C16—H16⋯O2^i^	0.93	2.53	3.4455 (17)	169

Symmetry code: (i) 
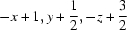
.

**Fig. 1.**
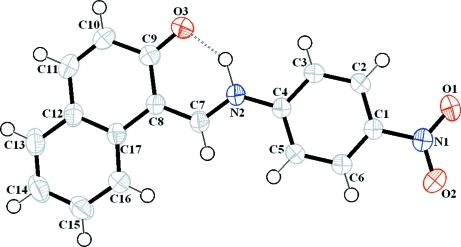
(Farrugia, 1997) The asymmetric unit of the title compound with the atomic labeling scheme. Displacement are drawn at the 50% probability level. Hydrogen bond shown as dashed line.

**Fig. 2.**
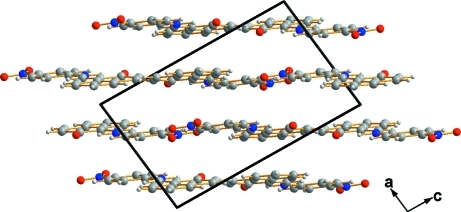
(Brandenburg, 2001) A diagram of the layered crystal packing in (I), viewed down the b axis, showing layers parallel to (101).

**Fig. 3.**
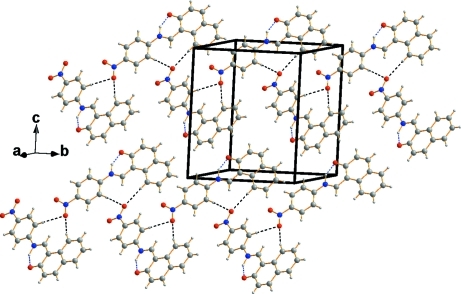
(Brandenburg, 2001) A part of crystal packing of (I) showing hydrogen bond connections in the same layer as dashed line.

## supplementary crystallographic information

### Comment

Schiff base compounds have been widely investigated over a century (Fan *et al.*, 2007; Kim *et al.*, 2005; Nimitsiriwat *et al.*, 2004). Some of the compounds have been found to have pharmaceutical and medicinal fields (Chen *et al.*, 1997; Ren *et al.*, 2002; Dao *et al.*, 2000; Sriram *et al.*, 2006; Karthikeyan *et al.*, 2006). They are also used as versatile ligands in coordination chemistry (Ali *et al.*, 2008; Kargar *et al.*, 2009; Yeap *et al.*, 2009).

As part of our ongoing studies of Schiff base complexes and derivatives we report here synthesis and the crystal structure of the title compound, obtained by the reaction of 2-hydroxy-1-naphthaldehyde with 4-nitroaniline, which crystallized in a zwitterionic form with cationic iminium and anionic naphtholate group.

The molecular structure of (I), and the atomic numbering used, is illustrated in Fig. 1. All bond distances and angles are within the ranges of accepted values (CSD, Allen, 2002) and in literature (Fun *et al.*, 2009; Nadeem *et al.*, 2009; Eltayeb *et al.*, 2008).

The main molecule is essentially planar with an rms deviation of 0.0350 Å, and the crystal structure exhibit alternating layers parallel to (101) plane (Fig. 2). In the crystal, molecules are linked *via* intermolecular C—H···O hydrogen bonds to form a two-dimensional layers parallel to (101) (Table 1, Fig. 3) and additional stabilization within these layers is provided by N—O···π and π···π stacking interactions. These interaction bonds link the molecules within the layers and also link the layers together and reinforcing the cohesion of the structure. An intramolecular N—H···O hydrogen bond occurs.

### Experimental

The title compound, (I), was prepared by refluxing a mixture of a solution containing (0.1 mmol) of 2-hydroxy-1-naphthaldehyde and (0.1 mmol) of 4-nitrobenzenamine in 20 ml methanol. The reaction mixture was stirred for 1 h under reflux. Microcrystals of (I) were obtained by allowing the clear solution to stand overnight. The powder product was dissolved and recrystallized from DMSO solution. Some red crystals were carefully isolated under polarizing microscope for analysis by x-ray diffraction.

### Refinement

H7 and H2N were located in difference Fourier maps and refined isotropically. The remaining H atoms were localized on Fourier maps but introduced in calculated positions and treated as riding on their parent atoms (C_aryl_) with C_aryl_—H_aryl_=0.95Å and *U*_iso_(H_aryl_)=1.2*U*_eq_(C_aryl_).

### Crystal data

**Table:**

C_17_H_12_N_2_O_3_	*F*(000) = 608
*M**_r_* = 292.29	*D*_x_ = 1.439 Mg m^−^^3^
Monoclinic, *P*2_1_/*c*	Mo *K*α radiation, λ = 0.71073 Å
Hall symbol: -P 2ybc	Cell parameters from 5160 reflections
*a* = 8.0503 (6) Å	θ = 3.0–30.1°
*b* = 12.8174 (9) Å	µ = 0.10 mm^−^^1^
*c* = 13.1833 (10) Å	*T* = 296 K
β = 97.271 (5)°	Needle, red
*V* = 1349.37 (17) Å^3^	0.15 × 0.06 × 0.04 mm
*Z* = 4	

### Data collection

**Table:**

Bruker SMART CCD area-detector diffractometer	3658 reflections with *I* > 2σ(*I*)
Radiation source: sealed tube	*R*_int_ = 0.074
graphite	θ_max_ = 39.3°, θ_min_ = 3.0°
φ and ω scans	*h* = −14→12
44074 measured reflections	*k* = −20→22
7946 independent reflections	*l* = −20→23

### Refinement

**Table:**

Refinement on *F*^2^	Primary atom site location: structure-invariant direct methods
Least-squares matrix: full	Secondary atom site location: difference Fourier map
*R*[*F*^2^ > 2σ(*F*^2^)] = 0.059	Hydrogen site location: inferred from neighbouring sites
*wR*(*F*^2^) = 0.190	H atoms treated by a mixture of independent and constrained refinement
*S* = 0.96	*w* = 1/[σ^2^(*F*_o_^2^) + (0.0963*P*)^2^] where *P* = (*F*_o_^2^ + 2*F*_c_^2^)/3
7946 reflections	(Δ/σ)_max_ = 0.001
207 parameters	Δρ_max_ = 0.53 e Å^−^^3^
0 restraints	Δρ_min_ = −0.26 e Å^−^^3^

### Special details

**Table:**

Geometry. All e.s.d.'s (except the e.s.d. in the dihedral angle between two l.s. planes) are estimated using the full covariance matrix. The cell e.s.d.'s are taken into account individually in the estimation of e.s.d.'s in distances, angles and torsion angles; correlations between e.s.d.'s in cell parameters are only used when they are defined by crystal symmetry. An approximate (isotropic) treatment of cell e.s.d.'s is used for estimating e.s.d.'s involving l.s. planes.
Refinement. Refinement of *F*^2^ against ALL reflections. The weighted *R*-factor *wR* and goodness of fit *S* are based on *F*^2^, conventional *R*-factors *R* are based on *F*, with *F* set to zero for negative *F*^2^. The threshold expression of *F*^2^ > σ(*F*^2^) is used only for calculating *R*-factors(gt) *etc*. and is not relevant to the choice of reflections for refinement. *R*-factors based on *F*^2^ are statistically about twice as large as those based on *F*, and *R*- factors based on ALL data will be even larger.

### Fractional atomic coordinates and isotropic or equivalent isotropic displacement parameters (Å^2^)

**Table:**

	*x*	*y*	*z*	*U*_iso_*/*U*_eq_	
O3	0.88419 (14)	−0.08384 (7)	0.31991 (8)	0.0392 (3)	
O2	0.44266 (15)	−0.40551 (8)	0.79254 (8)	0.0426 (3)	
O1	0.49601 (17)	−0.51766 (7)	0.67875 (9)	0.0500 (3)	
N2	0.74356 (13)	−0.11314 (7)	0.47852 (8)	0.0264 (2)	
N1	0.49499 (15)	−0.42784 (8)	0.71179 (9)	0.0320 (2)	
C17	0.82974 (14)	0.16828 (9)	0.44636 (9)	0.0234 (2)	
C8	0.82177 (14)	0.05641 (9)	0.42782 (9)	0.0234 (2)	
C6	0.55083 (16)	−0.24287 (9)	0.68580 (10)	0.0274 (2)	
H6	0.5057	−0.2275	0.7456	0.033*	
C1	0.55862 (16)	−0.34487 (9)	0.65258 (10)	0.0259 (2)	
C7	0.75161 (14)	−0.01080 (9)	0.49403 (10)	0.0245 (2)	
C2	0.62367 (17)	−0.37057 (9)	0.56335 (10)	0.0300 (3)	
H2	0.6267	−0.4396	0.5421	0.036*	
C12	0.90181 (15)	0.23306 (9)	0.37664 (10)	0.0273 (2)	
C16	0.76748 (16)	0.21715 (9)	0.52907 (10)	0.0293 (3)	
H16	0.721	0.1766	0.5768	0.035*	
C3	0.68369 (16)	−0.29201 (9)	0.50680 (10)	0.0290 (3)	
H3	0.7278	−0.3079	0.4468	0.035*	
C5	0.61127 (16)	−0.16388 (9)	0.62877 (10)	0.0265 (2)	
H5	0.6069	−0.0949	0.6501	0.032*	
C13	0.90891 (17)	0.34145 (10)	0.39091 (12)	0.0355 (3)	
H13	0.9569	0.383	0.3446	0.043*	
C10	0.96505 (18)	0.08390 (11)	0.27475 (11)	0.0352 (3)	
H10	1.0122	0.0574	0.2193	0.042*	
C4	0.67859 (14)	−0.18811 (9)	0.53937 (9)	0.0239 (2)	
C9	0.88945 (16)	0.01310 (9)	0.34053 (10)	0.0280 (2)	
C14	0.84615 (17)	0.38702 (10)	0.47216 (13)	0.0379 (3)	
H14	0.8517	0.459	0.4812	0.045*	
C15	0.77392 (17)	0.32417 (10)	0.54105 (12)	0.0346 (3)	
H15	0.7296	0.3547	0.5957	0.041*	
C11	0.96888 (17)	0.18704 (10)	0.29171 (11)	0.0336 (3)	
H11	1.0168	0.2302	0.2467	0.04*	
H7	0.704 (2)	0.0100 (13)	0.5544 (14)	0.043 (5)*	
H2N	0.798 (3)	−0.1327 (18)	0.4091 (18)	0.080 (7)*	

### Atomic displacement parameters (Å^2^)

**Table:**

	*U*^11^	*U*^22^	*U*^33^	*U*^12^	*U*^13^	*U*^23^
O3	0.0597 (6)	0.0256 (4)	0.0362 (5)	−0.0031 (4)	0.0217 (5)	−0.0042 (4)
O2	0.0637 (7)	0.0342 (5)	0.0348 (5)	−0.0030 (5)	0.0249 (5)	0.0036 (4)
O1	0.0847 (9)	0.0207 (4)	0.0502 (7)	−0.0069 (5)	0.0308 (6)	0.0002 (4)
N2	0.0325 (5)	0.0198 (4)	0.0283 (5)	−0.0011 (4)	0.0097 (4)	0.0016 (4)
N1	0.0419 (6)	0.0241 (5)	0.0322 (6)	−0.0016 (4)	0.0134 (5)	0.0031 (4)
C17	0.0224 (5)	0.0216 (5)	0.0260 (6)	−0.0003 (4)	0.0023 (4)	0.0025 (4)
C8	0.0253 (5)	0.0215 (5)	0.0235 (5)	−0.0003 (4)	0.0044 (4)	0.0014 (4)
C6	0.0332 (6)	0.0226 (5)	0.0282 (6)	−0.0006 (4)	0.0111 (5)	−0.0012 (4)
C1	0.0320 (6)	0.0206 (4)	0.0264 (6)	−0.0007 (4)	0.0089 (5)	0.0015 (4)
C7	0.0256 (5)	0.0212 (5)	0.0271 (6)	−0.0002 (4)	0.0052 (4)	0.0017 (4)
C2	0.0395 (7)	0.0196 (5)	0.0335 (6)	0.0000 (4)	0.0148 (5)	−0.0022 (4)
C12	0.0257 (5)	0.0236 (5)	0.0329 (6)	−0.0022 (4)	0.0054 (5)	0.0037 (4)
C16	0.0334 (6)	0.0251 (5)	0.0302 (6)	−0.0001 (5)	0.0073 (5)	−0.0012 (4)
C3	0.0369 (6)	0.0222 (5)	0.0306 (6)	−0.0001 (4)	0.0151 (5)	−0.0018 (4)
C5	0.0326 (6)	0.0193 (4)	0.0290 (6)	−0.0019 (4)	0.0097 (5)	−0.0026 (4)
C13	0.0334 (6)	0.0245 (5)	0.0499 (9)	−0.0044 (5)	0.0108 (6)	0.0053 (5)
C10	0.0471 (8)	0.0325 (6)	0.0291 (6)	−0.0030 (5)	0.0164 (6)	0.0009 (5)
C4	0.0268 (5)	0.0201 (4)	0.0259 (6)	−0.0012 (4)	0.0075 (4)	0.0005 (4)
C9	0.0334 (6)	0.0257 (5)	0.0259 (6)	−0.0012 (4)	0.0076 (5)	−0.0006 (4)
C14	0.0357 (7)	0.0220 (5)	0.0565 (9)	−0.0029 (5)	0.0079 (7)	−0.0021 (6)
C15	0.0367 (7)	0.0260 (6)	0.0413 (8)	0.0011 (5)	0.0059 (6)	−0.0060 (5)
C11	0.0382 (7)	0.0319 (6)	0.0329 (7)	−0.0054 (5)	0.0129 (6)	0.0046 (5)

### Geometric parameters (Å, °)

**Table:**

O3—C9	1.2714 (15)	C2—H2	0.93
O2—N1	1.2274 (14)	C12—C13	1.4021 (17)
O1—N1	1.2312 (14)	C12—C11	1.4304 (18)
N2—C7	1.3279 (15)	C16—C15	1.3810 (17)
N2—C4	1.3951 (14)	C16—H16	0.93
N2—H2N	1.09 (2)	C3—C4	1.4015 (16)
N1—C1	1.4504 (14)	C3—H3	0.93
C17—C16	1.4039 (16)	C5—C4	1.3931 (16)
C17—C12	1.4163 (15)	C5—H5	0.93
C17—C8	1.4546 (16)	C13—C14	1.372 (2)
C8—C7	1.3957 (15)	C13—H13	0.93
C8—C9	1.4450 (16)	C10—C11	1.3405 (18)
C6—C1	1.3827 (17)	C10—C9	1.4421 (17)
C6—C5	1.3855 (16)	C10—H10	0.93
C6—H6	0.93	C14—C15	1.395 (2)
C1—C2	1.3866 (16)	C14—H14	0.93
C7—H7	0.962 (19)	C15—H15	0.93
C2—C3	1.3763 (16)	C11—H11	0.93
			
C7—N2—C4	127.33 (10)	C17—C16—H16	119.3
C7—N2—H2N	109.9 (12)	C2—C3—C4	120.22 (11)
C4—N2—H2N	122.8 (12)	C2—C3—H3	119.9
O2—N1—O1	122.91 (11)	C4—C3—H3	119.9
O2—N1—C1	118.62 (10)	C6—C5—C4	119.81 (10)
O1—N1—C1	118.47 (10)	C6—C5—H5	120.1
C16—C17—C12	117.28 (11)	C4—C5—H5	120.1
C16—C17—C8	123.91 (10)	C14—C13—C12	120.93 (12)
C12—C17—C8	118.81 (10)	C14—C13—H13	119.5
C7—C8—C9	118.96 (10)	C12—C13—H13	119.5
C7—C8—C17	121.07 (10)	C11—C10—C9	121.53 (12)
C9—C8—C17	119.96 (10)	C11—C10—H10	119.2
C1—C6—C5	119.07 (10)	C9—C10—H10	119.2
C1—C6—H6	120.5	C5—C4—N2	123.17 (10)
C5—C6—H6	120.5	C5—C4—C3	120.04 (10)
C6—C1—C2	122.04 (11)	N2—C4—C3	116.79 (10)
C6—C1—N1	119.32 (10)	O3—C9—C10	119.44 (11)
C2—C1—N1	118.64 (10)	O3—C9—C8	122.68 (11)
N2—C7—C8	121.95 (11)	C10—C9—C8	117.87 (11)
N2—C7—H7	112.6 (10)	C13—C14—C15	119.19 (12)
C8—C7—H7	125.4 (10)	C13—C14—H14	120.4
C3—C2—C1	118.81 (11)	C15—C14—H14	120.4
C3—C2—H2	120.6	C16—C15—C14	120.79 (12)
C1—C2—H2	120.6	C16—C15—H15	119.6
C13—C12—C17	120.46 (11)	C14—C15—H15	119.6
C13—C12—C11	120.03 (11)	C10—C11—C12	122.29 (11)
C17—C12—C11	119.50 (11)	C10—C11—H11	118.9
C15—C16—C17	121.34 (11)	C12—C11—H11	118.9
C15—C16—H16	119.3		
			
C16—C17—C8—C7	−0.89 (19)	C1—C6—C5—C4	0.0 (2)
C12—C17—C8—C7	−179.83 (12)	C17—C12—C13—C14	−0.2 (2)
C16—C17—C8—C9	−179.95 (12)	C11—C12—C13—C14	−179.64 (14)
C12—C17—C8—C9	1.11 (18)	C6—C5—C4—N2	−179.26 (12)
C5—C6—C1—C2	−0.6 (2)	C6—C5—C4—C3	0.6 (2)
C5—C6—C1—N1	−179.78 (12)	C7—N2—C4—C5	−0.3 (2)
O2—N1—C1—C6	−3.2 (2)	C7—N2—C4—C3	179.85 (12)
O1—N1—C1—C6	176.80 (13)	C2—C3—C4—C5	−0.6 (2)
O2—N1—C1—C2	177.56 (13)	C2—C3—C4—N2	179.28 (12)
O1—N1—C1—C2	−2.4 (2)	C11—C10—C9—O3	177.94 (14)
C4—N2—C7—C8	179.22 (12)	C11—C10—C9—C8	−1.8 (2)
C9—C8—C7—N2	−0.77 (19)	C7—C8—C9—O3	1.8 (2)
C17—C8—C7—N2	−179.84 (11)	C17—C8—C9—O3	−179.16 (12)
C6—C1—C2—C3	0.6 (2)	C7—C8—C9—C10	−178.46 (12)
N1—C1—C2—C3	179.80 (12)	C17—C8—C9—C10	0.62 (19)
C16—C17—C12—C13	−0.20 (19)	C12—C13—C14—C15	−0.2 (2)
C8—C17—C12—C13	178.81 (12)	C17—C16—C15—C14	−1.4 (2)
C16—C17—C12—C11	179.27 (12)	C13—C14—C15—C16	1.0 (2)
C8—C17—C12—C11	−1.71 (18)	C9—C10—C11—C12	1.3 (2)
C12—C17—C16—C15	0.98 (19)	C13—C12—C11—C10	−179.98 (14)
C8—C17—C16—C15	−177.98 (13)	C17—C12—C11—C10	0.5 (2)
C1—C2—C3—C4	0.0 (2)		

### Hydrogen-bond geometry (Å, °)

**Table:**

*D*—H···*A*	*D*—H	H···*A*	*D*···*A*	*D*—H···*A*
N2—H2N···O3	1.09 (2)	1.57 (2)	2.5287 (15)	143 (2)
C5—H5···O2^i^	0.93	2.59	3.5136 (16)	173
C16—H16···O2^i^	0.93	2.53	3.4455 (17)	169

Symmetry codes: (i) −*x*+1, *y*+1/2, −*z*+3/2.
